# A Clinical Case

**Published:** 1887-04

**Authors:** A. B. Eadie

**Affiliations:** Toronto, Canadas


### A CLINICAL CASE.

A. B. Eadie, M. D., Toronto, Canada.

July 29th, 1886.—Mr. Mc. complains of attacks which recur about every ten or fourteen days, becoming more frequent of late, characterized as follows: They begin at night. Hot sweat for ten minutes, followed by chill for five minutes; these paroxysms follow one another without intermission for a night and a day, and terminate in vomiting of green, thick, and slimy matter, mixed with ingesta; during the attack his heart seems to beat in his back; his wife can feel it shake the bed. There is some dyspnœa.

He has had these attacks for *two years.* In the intervals the following conditions are present: Constipation; no stool for three days at a time; stool, although soft, is hard to pass and sometimes accompanied by nausea and ptyalism; he feels a burning heat all over internally; black and thick blood flows freely from least scratch on face; he feels as if the "blood flows to the head;" abdomen feels full all the time, is distended; can't bear the least touch of the finger over lumbar region on either side—right side worse; *percussion* here *quite impossible;* rumbling in abdomen like water moving; arteries throb; sleeps about three hours; wakes at half-past twelve A. M., and lies awake till morning; dozes an hour before rising; has *cramps in his hands, can't open them;* nervous, can't write; *has cramps in feet.*

Says his trouble "lies at pit of stomach;" rifts after eating; appetite soon sated, and stomach swells so that he can eat no more. Although he has been troubled for two years he has taken no medicine, hoping the attacks would wear away themselves, but now says he can stand it no longer, as they are growing worse.—℞ China$^{cm}$, four powders, one night and morning.

August 1st.—Reported better; feels like another man; says he never thought medicine could do so much good. Bowels seem all right; no difficulty in deficating; internal heat, formerly always present, has disappeared; nervousness better; still complains of rumbling in abdomen and sensitiveness to contact, etc. ℞ Sac. lac.

August 6th.—Reports continued improvement. ℞ China$^{cm}$, two powders, to be taken at intervals of seven days, with Sac. lac. between.

September 4th.—Called to say he continued well; tenderness in side all gone; no rumbling or abdominal trouble; no chill or fevers since.

January 23d, 1887.—Continues well; had once or twice some premonitory symptoms of the old fever attacks, but they proved abortive. He has had no attack since.

